# An HTS FP assay able to selectively identify STAT3-DBD inhibitors

**DOI:** 10.18632/oncotarget.26155

**Published:** 2018-10-02

**Authors:** Fiorella Meneghetti, Daniela Barlocco

**Affiliations:** Fiorella Meneghetti: Department of Pharmaceutical Sciences, University of Milan, Milano, Italy

**Keywords:** STAT3, signal transducer and activator of transcription, high-throughput, DNA-binding domain, fluorescence polarization

Over the last two decades, signal transducer and activator of transcription 3 (STAT3) signaling pathway has been confirmed to play a pivotal role in initiating and sustaining pathological processes, and its inhibitors are currently under investigation for the treatment of oncologic, hematologic and chronic inflammatory diseases [1]. During this time, the target of STAT3 drug discovery research has been strongly influenced by the clear relationship that phosphorylation of the Src homology 2 (SH2) domain significantly enhances homo-dimerization, and in turn increases translocation. This rational has led to the discovery and validation of a number of inhibitors able to abate STAT3 activity in a variety of settings. However, the targeting of the SH2 phosphotyrosine binding site by small molecule inhibitors has met with very limited success in a clinical setting [2, 3]. Since this failure could be attributed to the ability of STAT3 to translocate into the nucleus, and to be activated even in the absence of SH2 phosphorylation, Shih et al., in the paper published in this issue of Oncotarget, focused on inhibiting STAT3 DNA-binding domain (DBD) by targeting the DBD, as an alternative, and perhaps better, downstream target [4]. Nevertheless, the latter being a more challenging molecular target, only a limited amount of validated inhibitors have been taken forward. One of several significant hurdles associated with targeting STAT3/DNA binding is the lack of a well-validated high-throughput evaluation assay [4] for the identification of small molecules. Currently, only EMSA and ELISA-based methods reliably characterize STAT3 DBD inhibitors.

To close this gap and enhance research efforts into finding STAT3/DNA inhibitors, Shih et al. reported a new practical biophysical assay – the fluorescence polarization (FP) competition assay. The assay, which utilizes a truncated STAT3127-688 construct as a target and Bodipy-DNA as the probe [4], can be used to identify potential STAT3 DBD inhibitors, coined here as the STAT3127-688:DNA FP assay [4]. To ensure its validity, the assay was tested using both unlabelled consensus and non-consensus double-stranded DNA sequences. One interesting aspect noted by Shih et al. was that the equilibrium of STAT3127-688:DNA interaction was reached after 14 hr and was reliable up to 48 hr. This then allows one to understand time-dependent activities of inhibitors in a cell-free environment [4]. The pilot screening run by Shih et al. determined IC_50_ of known STAT3 DBD small molecule inhibitors in a 96-well plate format; for compounds inS3-54, inS3-54A18 and niclosamide they were 21.3 ± 6.9 μM, 126 ± 39.7 μM and 219 ± 43.4 μM after 24 hr, respectively.

Interesting to note, Shih et al. showed that the STAT3127-688:DNA FP assay can be conducted in parallel with another FP assay [4] designed to identify STAT3 SH2 domain inhibitors, as reported by Schust and Berg [5], thus helping to distinguish between the different mode(s) of action of an inhibition. As several of the available inhibitors evidenced a dual mechanism of action, this test, when undertaken concurrently in two halves of the same plate, identifies the relative contribution of each target domain. This can allow a more reliable selection between ligand’s alternative mode(s) of action within screening libraries.

In particular, the cell-free method described by this research team, if functional for Platinum-complex inhibitors which are recognized to alter the biological function of different targets [6], would be very helpful to rule out effects on upstream proteins or off-target effects.

Looking ahead, the STAT3127-688:DNA FP assay published by Shih et al. will aid in the bioactivity evaluation of STAT3 inhibitors *in vitro*, and will be extended to screen, with a simple protocol, molecules interacting with other members of the STAT family, including STAT1 and STAT5, both valid protein targets for drug discovery [4].
